# P-675. Chronic Conditions as Risk Factors for Respiratory Syncytial Virus-Associated Hospitalization among Community-Dwelling Adults Aged 18-49 Years, 2016-2017 and 2017-2018 Seasons

**DOI:** 10.1093/ofid/ofaf695.888

**Published:** 2026-01-11

**Authors:** Rebecca C Woodruff, Gordana Derado, Pam Daily Kirley, Lucy S Witt, Patricia A Ryan, Ruth Lynfield, Fiona Keating, Kevin Popham, Ann Thomas, William Schaffner, Sarah Hamid, Huong Pham, Christopher A Taylor, Fiona P Havers, Michael Melgar

**Affiliations:** Centers for Disease Control and Prevention, Chamblee, Georgia; CDC, Atlanta, Georgia; California Emerging Infections Program, Oakland, California; Emory University, Atlanta, Georgia; Maryland Department of Health, Baltimore, Maryland; Minnesota Department of Health, St. Paul, MN; New York State Department of Health, Albany, New York; Universirty of Rochester, Rochester, NY; Oregon Health Authority, Portland, Oregon; Vanderbilt University Medical Center, Nashville, Tennessee; Epidemic Intelligence Service, Centers for Disease Control and Prevention; Coronavirus and other Respiratory Viruses Division, National Center for Immunization and Respiratory Disease, CDC, Atlanta, Georgia; Centers for Disease Control and Prevention, Chamblee, Georgia; Centers for Disease Control and Prevention, Chamblee, Georgia; Centers for Disease Control and Prevention, Chamblee, Georgia; Centers for Disease Control and Prevention, Chamblee, Georgia

## Abstract

**Background:**

Data on chronic conditions associated with increased respiratory syncytial virus (RSV)-associated hospitalization rates among adults aged ≥ 50 years have guided RSV vaccination recommendations. Similar data are needed for younger adults.

**Figure:**
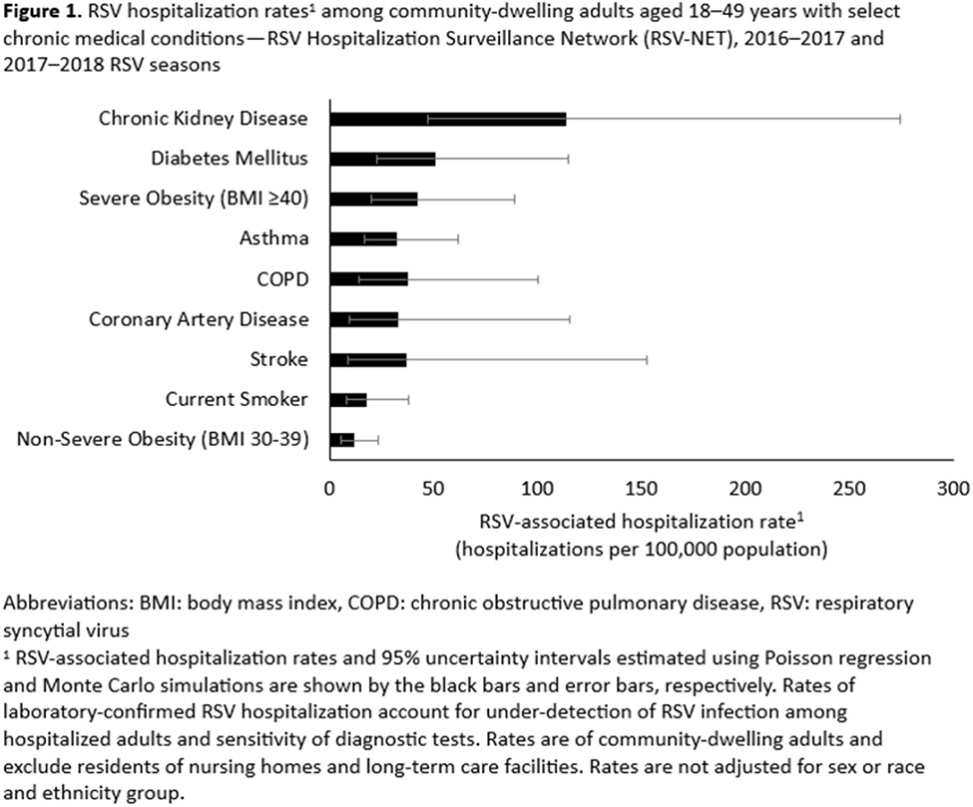

**Figure:**
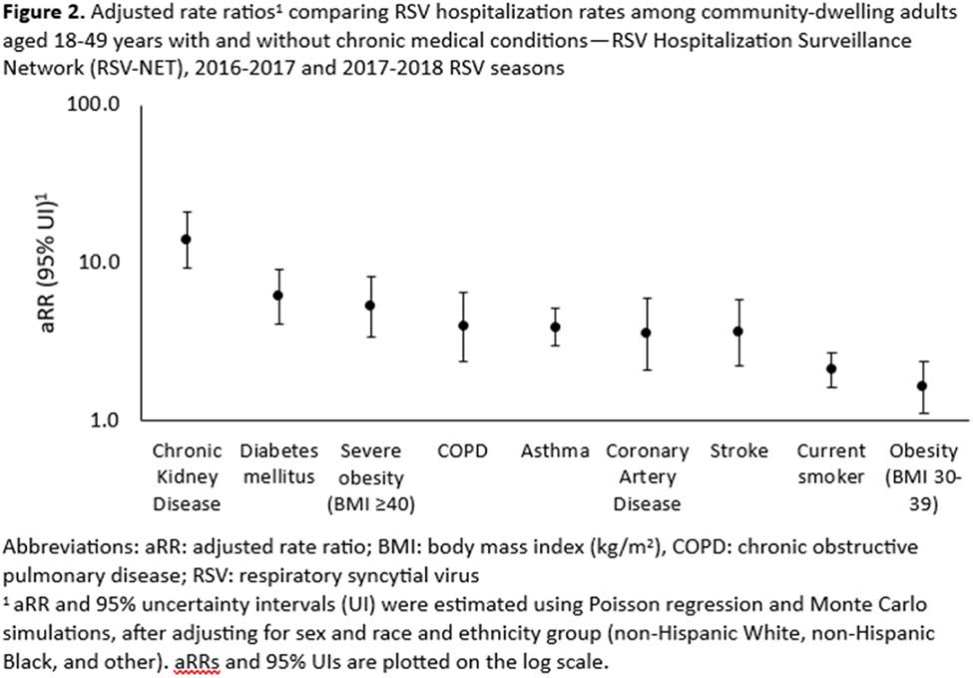

**Methods:**

We compared RSV hospitalization rates among community-dwelling adults aged 18-49 years with and without nine chronic medical conditions in a 38-county catchment area across seven states. Numerators included adults with and without each chronic condition who were hospitalized with laboratory-confirmed RSV infection identified through the RSV Hospitalization Surveillance Network (RSV-NET) during two RSV surveillance seasons during 2016-2018. Denominators were catchment area population estimates of adults with and without self-reported history of each chronic condition from the Behavioral Risk Factor Surveillance System and the US Census. Poisson regression using Monte Carlo simulation generated unadjusted rates and adjusted rate ratios (aRR) and 95% Monte Carlo uncertainty intervals (UI), adjusted for sex and race or ethnicity group.

**Results:**

Among community-dwelling adults aged 18-49 years, RSV hospitalization rates ranged from 11.7 hospitalizations per 100,000 (UI: 5.9, 23.6) for adults with non-severe obesity (body mass index [BMI] 30-39 kg/m^2^) to 113.1 hospitalizations per 100,000 (UI: 47.3, 274.3) for adults with chronic kidney disease (Figure 1). Those with each of the nine chronic conditions had higher RSV hospitalization rates compared to those without the respective conditions: chronic kidney disease (aRR=13.9, UI: 9.2, 21.2), diabetes (aRR=6.1, UI: 4.1, 9.1), severe obesity (BMI ≥ 40 kg/m^2^; aRR=5.3, UI: 3.4, 8.3), chronic obstructive pulmonary disease (aRR=4.0, UI: 2.4, 6.5), asthma (aRR=3.9, UI: 3.0, 5.1), coronary artery disease (aRR=3.6, UI: 2.1, 6.0), stroke (aRR=3.6, CI: 2.3, 5.9), current smoking (aRR=2.1, UI: 1.6, 2.7), and non-severe obesity (aRR=1.6, UI: 1.1, 2.4) (Figure 2). RSV hospitalization rates were higher among adults aged 18-49 years with 1 (aRR=2.2, UI: 1.7, 2.9) or ≥2 chronic conditions (aRR=8.3, UI: 6.4-10.9) vs. none.

**Conclusion:**

Chronic conditions were associated with higher rates of RSV hospitalization among younger adults, which can guide national vaccination recommendations.

**Disclosures:**

Lucy S. Witt, MD, MPH, Merck & Co: Grant/Research Support William Schaffner, MD, Abbott Dignostics: Honoraria

